# Computational models of dopamine release measured by fast scan cyclic voltammetry in vivo

**DOI:** 10.1093/pnasnexus/pgad044

**Published:** 2023-02-10

**Authors:** N Shashaank, Mahalakshmi Somayaji, Mattia Miotto, Eugene V Mosharov, Emily A Makowicz, David A Knowles, Giancarlo Ruocco, David L Sulzer

**Affiliations:** Department of Computer Science, Columbia University, New York, NY 10027, USA; New York Genome Center, New York, NY 10013, USA; Division of Molecular Therapeutics, New York State Psychiatric Institute, New York, NY 10032, USA; Division of Molecular Therapeutics, New York State Psychiatric Institute, New York, NY 10032, USA; Department of Psychiatry, Columbia University, New York, NY 10032, USA; Department of Physics, Sapienza University, Rome 00185, Italy; Center for Life Nano & Neuroscience, Italian Institute of Technology, Rome 00161, Italy; Division of Molecular Therapeutics, New York State Psychiatric Institute, New York, NY 10032, USA; Department of Psychiatry, Columbia University, New York, NY 10032, USA; Department of Neurology, Columbia University, New York, NY 10032, USA; Division of Molecular Therapeutics, New York State Psychiatric Institute, New York, NY 10032, USA; Department of Neuroscience, Columbia University, New York, NY 10032, USA; Department of Computer Science, Columbia University, New York, NY 10027, USA; New York Genome Center, New York, NY 10013, USA; Department of Systems Biology, Columbia University, New York, NY 10032, USA; Data Science Institute, Columbia University, New York, NY 10027, USA; Department of Physics, Sapienza University, Rome 00185, Italy; Center for Life Nano & Neuroscience, Italian Institute of Technology, Rome 00161, Italy; Division of Molecular Therapeutics, New York State Psychiatric Institute, New York, NY 10032, USA; Department of Psychiatry, Columbia University, New York, NY 10032, USA; Department of Neurology, Columbia University, New York, NY 10032, USA; Department of Pharmacology, Columbia University, New York, NY 10032, USA

**Keywords:** computational neuroscience, dopamine, fast-scan cyclic voltammetry, synaptic transmission

## Abstract

Dopamine neurotransmission in the striatum is central to many normal and disease functions. Ventral midbrain dopamine neurons exhibit ongoing tonic firing that produces low extrasynaptic levels of dopamine below the detection of conventional extrasynaptic cyclic voltammetry (∼10–20 nanomolar), with superimposed bursts that can saturate the dopamine uptake transporter and produce transient micromolar concentrations. The bursts are known to lead to marked presynaptic plasticity via multiple mechanisms, but analysis methods for these kinetic parameters are limited. To provide a deeper understanding of the mechanics of the modulation of dopamine neurotransmission by physiological, genetic, and pharmacological means, we present three computational models of dopamine release with different levels of spatiotemporal complexity to analyze in vivo fast-scan cyclic voltammetry recordings from the dorsal striatum of mice. The models accurately fit to cyclic voltammetry data and provide estimates of presynaptic dopamine facilitation/depression kinetics and dopamine transporter reuptake kinetics, and we used the models to analyze the role of synuclein proteins in neurotransmission. The models’ results support recent findings linking the presynaptic protein α-synuclein to the short-term facilitation and long-term depression of dopamine release, as well as reveal a new role for β-synuclein and/or γ-synuclein in the long-term regulation of dopamine reuptake.

**Significance Statement**

Electrochemical detection methods such as fast-scan cyclic voltammetry and amperometry are widely used to measure dopamine release in the brain. However, these methods are limited in their ability to quantify the kinetics of dopamine that affect synaptic transmission. In this work, we developed three computational models that can closely fit in vivo fast-scan cyclic voltammetry traces of dopamine release recorded in the dorsal striatum. These models provide biologically interpretable parameters of dopamine release and reuptake and will enable researchers to better quantify the effects of various neuronal proteins and pharmacological inhibitors on striatal extrasynaptic dopamine concentration.

## Introduction

Dopamine (DA) is a neurotransmitter that plays important roles in learning and memory, drug and alcohol addiction, attention deficit disorder, schizophrenia, and neurodegenerative disorders including Parkinson's disease (1). Electrochemical recording methods, particularly fast-scan cyclic voltammetry (FSCV) and constant potential amperometry, are used to record and analyze evoked DA release from the axons of dopamine neurons in rodent brains in vivo (2, 3), ex vivo in brain slice preparations (4, 5), and in cultured neurons (6, 7). The development of computational models to quantitatively describe FSCV and amperometry recordings of evoked DA release (8–13) has enabled the detailed analysis of DA release and reuptake kinetics under different stimulation paradigms both ex vivo and in vivo. However, the plasticity of DA neurotransmission is far more complex in vivo due to multiple factors that are less important in culture or slice preparations, including modulatory inputs from neurotransmitters such as acetylcholine and γ-aminobutyric acid (GABA) and multiple presynaptic regulatory proteins such as the synapsins and synucleins. Importantly, ventral midbrain DA neurons are tonic pacemakers, but their cell bodies are absent in slice preparations.

In vivo, tonic firing produces minimal DA levels in the striatum and is interspersed with burst-firing activity that can cause DA release to saturate the dopamine transporter (DAT) reuptake system, thus providing a nonlinear increase in extracellular DA during bursts (14). As reported in a study of spontaneous DA release (9), the magnitude and duration of burst-firing-evoked DA release in vivo are subject to multiple depressing and facilitatory influences that remain mostly uncharacterized. As such, deciphering the kinetics of DA release during burst firing is central for elucidating the interactions of DA in vivo and providing insights into the mechanisms responsible for DA release and regulation in normal development and function, exposure to drugs (ranging from antipsychotics to recreational/abused drugs), and many disease states. We have created three computational models of burst-firing striatal DA release in vivo, each of which closely fits the data and can be used to derive biologically interpretable parameters such as estimates of facilitation/depression kinetics, amount of DA release, and DAT reuptake kinetics. These models account for multiple biological, electrochemical, and experimental factors that affect the FSCV trace and can be adapted for other approaches to detect extracellular DA (15) and for analyzing release kinetics of other neurotransmitters.

**Fig. 1.**
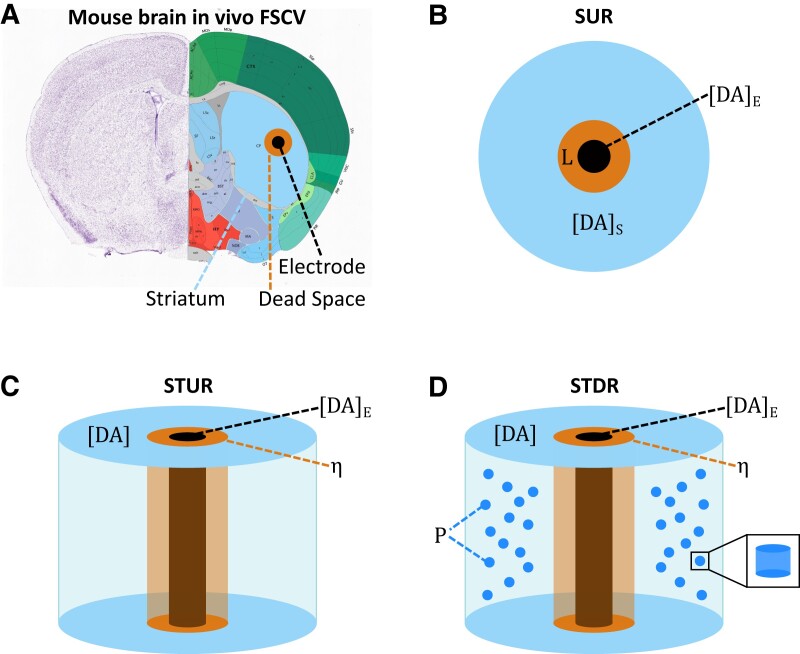
Schematic representations of computational models. A, Coronal slice of the mouse brain (adopted from the Allen Reference Atlas - Mouse Brain (18)). During in vivo FSCV experiments, a bipolar electrode in the ventral midbrain (not shown) is used to electrically stimulate the cell bodies of dopaminergic neurons, causing DA release via vesicular exocytosis from dopaminergic axons in the dorsal striatum (blue). The DA molecules diffuse to the carbon-fiber electrode (black) through striatal tissue and an area of damaged tissue known as the “dead space” (orange) that is formed when the electrode is inserted into the brain. B, Simple Uniform Release Model. DA release sites are modeled as a continuous release source within the striatum using temporal diffusion in one dimension without calculating the spatial characteristics of the striatum. C, Spatiotemporal Uniform Release Model, which is similar to the Simple Uniform Release model, except that the spatial characteristics of the dorsal striatum are modeled as a cylinder using isotropic diffusion in two dimensions. D, Spatiotemporal Discrete Release Model, which is similar to the Spatiotemporal Uniform Release model, with the addition of release sites modeled as discrete point sources positioned throughout the striatum.

The models simulate the interactions between extracellular DA released into striatal tissue, the carbon-fiber electrode that detects DA, and an intermediate area of damaged tissue known as the “dead space,” formed when inserting the electrode into the tissue (16) (Fig. 1A). When an electrical stimulus is applied, DA released by vesicular exocytosis diffuses through the extracellular space and dead space until it reaches the carbon-fiber electrode. In contrast to amperometry electrodes that “consume” DA molecules through oxidation, FSCV electrodes act as reflective surfaces that do not consume DA molecules and cause them to diffuse back into the tissue through the dead space (5). As a result, the concentration of extracellular DA in the striatum with FSCV experiments is mostly regulated by DAT uptake. The concentration of DA measured at the electrode is further altered by electrochemical adsorption (17), which causes the signal baseline to increase when multiple bursts of stimuli are triggered in quick succession. Our models can also be used to simulate DA release and diffusion without the recording “artifacts” introduced by the electrode, dead space, and adsorption (Fig. S1).

While the three models described here differ in the implementation of release and diffusion mechanics, their functional predictions are very similar. The *Simple Uniform Release* (SUR) model (Fig. 1B) is one-dimensional and computes DA release as a uniform distribution in the striatum that diffuses toward the carbon-fiber electrode without taking into account physical characteristics of the striatum. The *Spatiotemporal Uniform Release* (STUR) model (Fig. 1C) is two-dimensional and incorporates spatiotemporal diffusion of DA in the striatum using cylindrical coordinates and a uniform release distribution similar to the SUR model. The *Spatiotemporal Discrete Release* (STDR) model (Fig. 1D) is similar to the STUR model but uses discrete DA release sites positioned throughout the striatum. The mathematical equations for these models are described in the Materials and Methods section.

Our models contribute to the current state-of-the-art research in four key aspects. First, our models describe plasticity kinetics of evoked DA release for in vivo FSCV traces from anesthetized mice, in contrast to a similar study primarily focused on awake mice (9). Second, the spatiotemporal models provide mathematical equations for discrete release sites and the dead space, whereas prior studies (10, 11) mention these features without equations. Third, the creation of three computational models with different release and diffusion mechanics gives researchers the flexibility to choose the model that best captures their specific experimental conditions. Finally, we provide a statistical inference algorithm known as automatic differentiation variational inference (ADVI) (19, 20) for researchers to fit the adjustable parameters (with associated uncertainty estimates) in the models based on biological constraints.

## Results

**Fig. 2.**
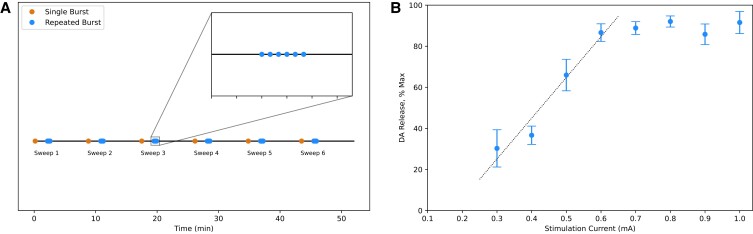
In vivo fast-scan cyclic voltammetry. A, Stimulus protocol for measuring plasticity kinetics in FSCV experiments. The experimental protocol is comprised of six sweeps, and each sweep contains a “Single Burst” and “Repeated Burst” protocol, with a 2-min recovery time between protocols and a 6-min recovery time between sweeps. A Single Burst (orange) is comprised of 30 pulses at 50 Hz, while a Repeated Burst (blue) is a series of six Single Bursts with 5 s between each burst. B, Stimulation Current vs. Evoked DA Release. In vivo FSCV experiments were conducted in the dorsal striatum of WT mice, with electrical bursts applied at 50 Hz, 30 pulses and stimulation currents ranging from 0.1–1 mA. Blue dots denote averaged evoked DA release resulting from the electrical bursts (*N* = 6 animals), and error bars report SEM. A linear relationship between stimulation current and evoked DA release is visible between 0.3–0.6 mA (*R*^2^ = 0.95), before DA release starts to saturate beyond 0.6 mA. DA release was undetectable at 0.1 and 0.2 mA.

We fit the computational models to in vivo recordings of dorsal striatum DA release evoked by multiple burst stimuli in the ventral midbrain (21). The complete list of model parameter estimates is provided in the Supplementary Information (Tables S1, S2, S3). Briefly, the experimental procedure was comprised of 6 “Single Burst” and “Repeated Burst” stimulation protocols (each pass being termed a “sweep”), with a 2-min recovery time between protocols and a 6-min recovery time between sweeps (Fig. 2A). The Single Burst protocol was a train of 30 pulses at 50 Hz, and the Repeated Burst protocol was a series of six Single Bursts with a 5 s interstimulus period between two bursts. The relationship between number of pulses and pulse frequency to DA release is linear with these stimulus parameters (21), as well as the relationship between stimulation current and DA release (Fig. 2B).

### Three kinetic components are sufficient to describe DA release kinetics in wildtype mice in vivo

**Fig. 3.**
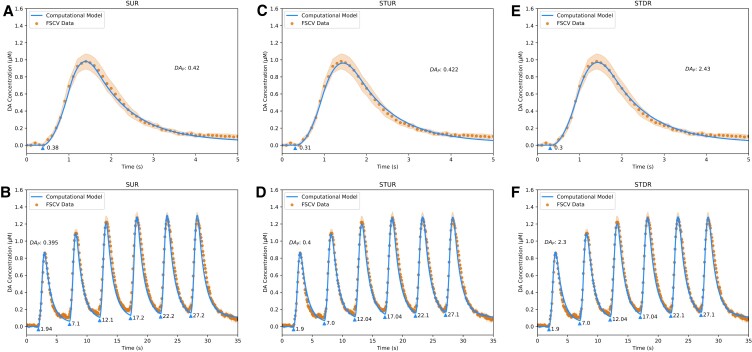
Fits of DA computational models to sweep 1 FSCV traces from WT mice in dorsal striatum. A, fit of Simple Uniform Release Model (*R*^2^ = 0.99) to Single Burst protocol. B, fit of Simple Uniform Release Model (*R*^2^ = 0.96) to Repeated Burst protocol. C, fit of Spatiotemporal Uniform Release Model (*R*^2^ = 0.99) to Single Burst protocol. D, fit of Spatiotemporal Uniform Release Model (*R*^2^ = 0.98) to Repeated Burst protocol. E, fit of Spatiotemporal Discrete Release Model (*R*^2^ = 0.99) to Single Burst protocol. F, fit of Spatiotemporal Discrete Release Model (*R*^2^ = 0.98) to Repeated Burst protocol. Blue lines are model fits, blue triangles indicate start times *t*_*i*_ of electrical bursts, orange dots are averaged FSCV traces (*N* = 7 animals), and orange ribbons report SEM.

**Fig. 4.**
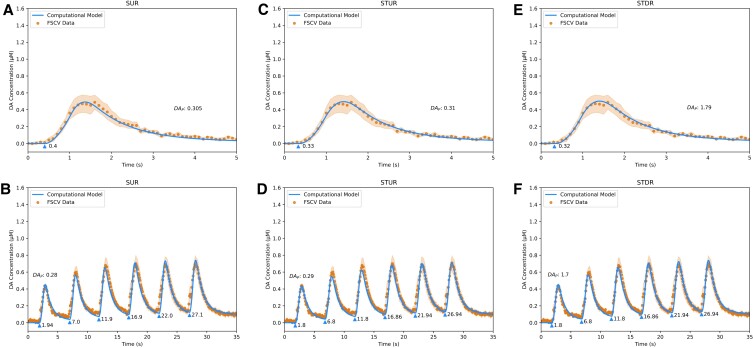
Fits of DA computational models to sweep 6 FSCV traces from WT mice in dorsal striatum. A, fit of Simple Uniform Release Model (*R*^2^ = 0.98) to Single Burst protocol. B, fit of Simple Uniform Release Model (*R*^2^ = 0.93) to Repeated Burst protocol. C, fit of Spatiotemporal Uniform Release Model (*R*^2^ = 0.99) to Single Burst protocol. D, fit of Spatiotemporal Uniform Release Model (*R*^2^ = 0.95) to Repeated Burst protocol. E, fit of Spatiotemporal Discrete Release Model (*R*^2^ = 0.99) to Single Burst protocol. F, fit of Spatiotemporal Discrete Release Model (*R*^2^ = 0.96) to Repeated Burst protocol. Blue lines are model fits, blue triangles indicate start times *t*_*i*_ of electrical bursts, orange dots are averaged FSCV traces (*N* = 7 animals), and orange ribbons report SEM.

To analyze the kinetics of DA release under normal conditions, the models were fit to FSCV data from wildtype (WT) mice using the Single Burst and Repeated Burst protocols from Sweep 1 (Fig. 3) and Sweep 6 (Fig. 4) of the experimental procedure. Consistent with conclusions from a study of spontaneous DA release kinetics in behaving mice (9), the complex dynamics of the data require three kinetic components to capture changes in DA release over time: short-term facilitation, short-term depression, and long-term depression. Each kinetic is described in the models by a “plasticity factor” *p*_*j*_ that controls the facilitative/depressive effect's magnitude and a time constant *τ*_*j*_ that controls the kinetic's duration (see Materials and Methods for more information). The kinetic parameter estimates (Table 1) for the Single Burst and Repeated Burst protocols indicate that in WT mice, the plasticity factor for short-term facilitation is 3.5-fold larger than short-term depression, while the time constant of short-term depression is ∼1.7-fold to 2-fold longer than short-term facilitation.

**Table 1.** Best-fit model parameters for DA release kinetics and DAT activity. For all fits presented in the Results section, *K*_*m*_ was set to 0.2 μΜ.

	Short-term facilitation		Short-term depression		Long-term depression		Burst type	SUR model		STUR model		STDR model	
	*p*_1_	*τ*_1_ (s)	*p*_2_	*τ*_2_ (s)	*p*_3_	*τ*_3_ (s)		*DA*_*P*_ (μM/mA)	*V*_*m*_ (μM/s)	*DA*_*P*_ (μM/mA)	*V*_*m*_ (μM/s)	*DA*_*P*_ (μM×μm/mA)	*V*_*m*_ (μM/s)
**WT** **Sweep 1**	0.0105	7.50	−0.003	15.0	−0.0011	900	**S**	0.420	4.8	0.422	4.8	2.43	4.8
							**R**	0.395	4.8	0.400	4.8	2.30	4.8
**WT** **Sweep 6**	0.0105	7.50	−0.003	12.5	−0.0011	900	**S**	0.305	3.2	0.310	3.2	1.79	3.2
							**R**	0.280	3.2	0.290	3.2	1.70	3.2
**α-Syn ΚΟ** **Sweep 1**	0.0050	7.25	−0.003	37.5	0	900	**S**	0.460	4.8	0.460	4.8	2.65	4.8
							**R**	0.450	4.8	0.443	4.8	2.55	4.8
**α-Syn ΚΟ** **Sweep 6**	0.0040	7.25	−0.003	45.0	0	900	**S**	0.320	3.2	0.315	3.2	1.80	3.2
							**R**	0.295	3.2	0.295	3.2	1.70	3.2
**Syn ΤΚΟ** **Sweep 1**	0.0040	7.25	−0.003	37.5	0	900	**S**	0.460	4.8	0.460	4.8	2.63	4.8
							**R**	0.450	4.8	0.450	4.8	2.55	4.8
**Syn ΤΚΟ** **Sweep 6**	0.0040	7.25	−0.004	37.5	0	900	**S**	0.506	5.6	0.505	5.6	2.77	5.6
							**R**	0.467	5.6	0.467	5.6	2.65	5.6

The long-term depression kinetic contributes to an ∼50% decrease in evoked DA release observed in WT mice between Sweeps 1 and 6. While the plasticity factor of long-term depression is weaker than both short-term kinetics (Table 1), the time constant of 15 min is ∼60-fold longer than the closest time constant for short-term kinetics, consistent with prior literature (9, 22). The release parameter estimates (Table 1) indicate that DAT activity *V*_*m*_ and DA release *DA*_*P*_ decrease between Sweeps 1 and 6 by 33.3% and ∼25–30%, respectively. Further experiments might investigate the biological mechanisms that relate to long-term plasticity of release and reuptake that could account for these effects, such as synaptic exhaustion at vesicular release sites and DAT endocytosis.

### Synuclein proteins regulate DA release kinetic components in vivo

In order to determine how the kinetic components can be altered in vivo, we analyzed the impact of the synuclein family of neuronal proteins on DA release. There are three genetically distinct synuclein proteins: α-synuclein (α-Syn), β-synuclein (β-Syn), and γ-synuclein (γ-Syn) (23). α-Syn and β-Syn are expressed in brain regions including the striatum, thalamus, hippocampus, neocortex, and cerebellum (24), and γ-Syn is expressed in the peripheral nervous system as well as the brain (25). α-Syn has been associated with disease-related protein aggregates in Parkinson's disease and other neurodegenerative disorders, β-Syn has been shown to inhibit α-Syn aggregation, and abnormal levels of γ-Syn have been linked to multiple cancers (26). Although the normal physiological functions of the synucleins are still under study, they regulate evoked DA release both in the striatal slice (27) and in vivo (21), likely by modulating steps in synaptic vesicle recycling and exocytosis (28, 29).

**Fig. 5.**
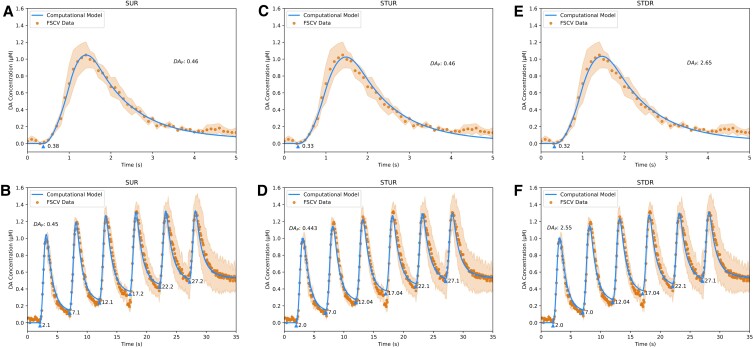
Fits of DA computational models to sweep 1 FSCV traces from α-Syn KO mice in dorsal striatum. A, fit of Simple Uniform Release Model (*R*^2^ = 0.99) to Single Burst protocol. B, fit of Simple Uniform Release Model (*R*^2^ = 0.97) to Repeated Burst protocol. C, fit of Spatiotemporal Uniform Release Model (*R*^2^ = 0.98) to Single Burst protocol. D, fit of Spatiotemporal Uniform Release Model (*R*^2^ = 0.97) to Repeated Burst protocol. E, fit of Spatiotemporal Discrete Release Model (*R*^2^ = 0.98) to Single Burst protocol. F, fit of Spatiotemporal Discrete Release Model (*R*^2^ = 0.97) to Repeated Burst protocol. Blue lines are model fits, blue triangles indicate start times *t*_*i*_ of electrical bursts, orange dots are averaged FSCV traces (*N* = 3 animals), and orange ribbons report SEM.

**Fig. 6.**
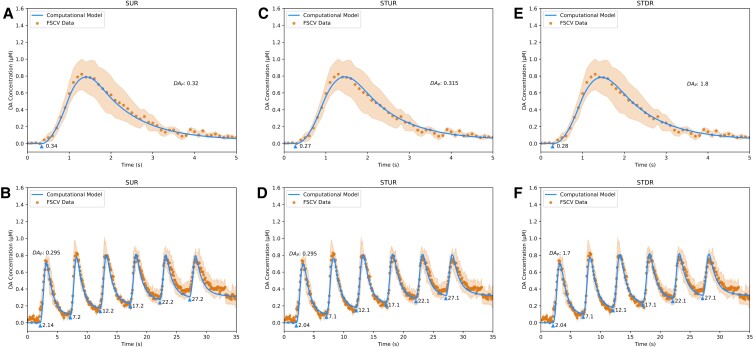
Fits of DA computational models to sweep 6 FSCV traces from α-Syn KO mice in dorsal striatum. A, fit of Simple Uniform Release Model (*R*^2^ = 0.99) to Single Burst protocol. B, fit of Simple Uniform Release Model (*R*^2^ = 0.94) to Repeated Burst protocol. C, fit of Spatiotemporal Uniform Release Model (*R*^2^ = 0.99) to Single Burst protocol. D, fit of Spatiotemporal Uniform Release Model (*R*^2^ = 0.96) to Repeated Burst protocol. E, fit of Spatiotemporal Discrete Release Model (*R*^2^ = 0.99) to Single Burst protocol. F, fit of Spatiotemporal Discrete Release Model (*R*^2^ = 0.96) to Repeated Burst protocol. Blue lines are model fits, blue triangles indicate start times *t*_*i*_ of electrical bursts, orange dots are averaged FSCV traces (*N* = 3 animals), and orange ribbons report SEM.

**Fig. 7.**
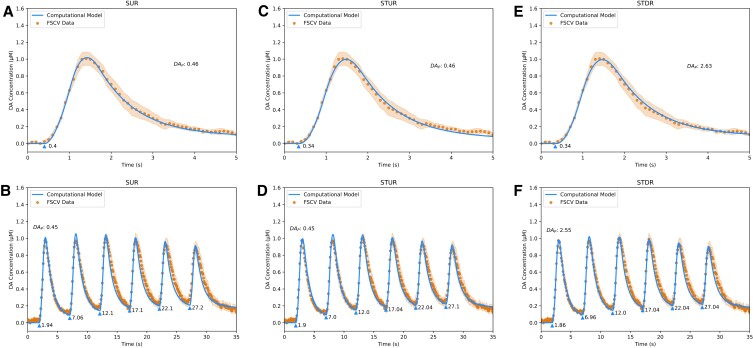
Fits of DA computational models to sweep 1 FSCV traces from Syn TKO mice in dorsal striatum. A, fit of Simple Uniform Release Model (*R*^2^ = 1.00) to Single Burst protocol. B, fit of Simple Uniform Release Model (*R*^2^ = 0.95) to Repeated Burst protocol. C, fit of Spatiotemporal Uniform Release Model (*R*^2^ = 0.99) to Single Burst protocol. D, fit of Spatiotemporal Uniform Release Model (*R*^2^ = 0.97) to Repeated Burst protocol. E, fit of Spatiotemporal Discrete Release Model (*R*^2^ = 1.00) to Single Burst protocol. F, fit of Spatiotemporal Discrete Release Model (*R*^2^ = 0.97) to Repeated Burst protocol. Blue lines are model fits, blue triangles indicate start times *t*_*i*_ of electrical bursts, orange dots are averaged FSCV traces (*N* = 7 animals), and orange ribbons report SEM.

**Fig. 8.**
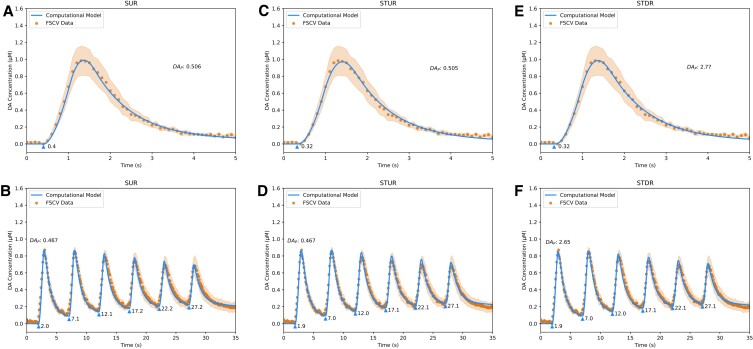
Fits of DA computational models to sweep 6 FSCV traces from Syn TKO mice in dorsal striatum. A, fit of Simple Uniform Release Model (*R*^2^ = 1.00) to Single Burst protocol. B, fit of Simple Uniform Release Model (*R*^2^ = 0.96) to Repeated Burst protocol. C, fit of Spatiotemporal Uniform Release Model (*R*^2^ = 0.99) to Single Burst protocol. D, fit of Spatiotemporal Uniform Release Model (*R*^2^ = 0.98) to Repeated Burst protocol. E, fit of Spatiotemporal Discrete Release Model (*R*^2^ = 1.00) to Single Burst protocol. F, fit of Spatiotemporal Discrete Release Model (*R*^2^ = 0.98) to Repeated Burst protocol. Blue lines are model fits, blue triangles indicate start times *t*_*i*_ of electrical bursts, orange dots are averaged FSCV traces (*N* = 7 animals), and orange ribbons report SEM.

To analyze the interactions of synucleins on DA release in vivo and its effects on the kinetics of DA release, the models were fit to FSCV data from two lines of mutant mice: α-Syn knockout (KO) mice (Figs. 5 and 6) that are deficient in α-Syn, and synuclein triple knockout (Syn TKO) mice (Figs. 7 and 8) that are deficient in α-Syn, β-Syn, and γ-Syn. Like WT mice, the data were taken from the Single Burst and Repeated Burst protocols for Sweep 1 and Sweep 6. Comparing the models’ kinetic parameter estimates for α-Syn KO mice (Table 1) revealed that the plasticity factor of short-term facilitation decreased by ∼40–50%, indicating that α-Syn expression enhances short-term facilitation. Additionally, the time constant of short-term depression increased by ∼2.5-fold, indicating that α-Syn expression slows down the kinetics of short-term depression. Finally, the plasticity factor of long-term depression is absent, implying that long-term depression is fully dependent on α-Syn expression. Similar changes in facilitation/depression constants were observed in Syn TKO mice, indicating that the effect was not related to β-Syn or γ-Syn expression. These results reinforce conclusions by Somayaji et al. (21) that α-Syn controls the short-term facilitation and long-term depression of DA release.

Similar to WT mice, the models’ release parameter estimates (Table 1) for α-Syn KO mice indicate a 33.3% decrease in DAT activity and ∼30–35% decrease in DA release between Sweeps 1 and 6, which accounts for the smaller ∼25% decrease in evoked DA release observed in the data between the sweeps in the absence of any long-term depression kinetic. In contrast, the models’ estimates for the Syn TKO mice indicate a relatively small *increase* in both DAT activity and DA release between Sweeps 1 and 6 by 16.7% and ∼5–10%, respectively (Table 1). This suggests a previously unknown role of β-Syn and/or γ-Syn in the long-term regulation of DAT uptake and vesicular DA release and may in part underlie the observation that the Syn TKO line has a greater effect than the α-Syn KO line on exocytosis of peptides from chromaffin cells and cultured hippocampal neurons (30).

## Discussion

In this paper, we presented three computational models of DA release to fit and analyze FSCV recordings of evoked DA release in the dorsal striatum of anesthetized mice in vivo. Using these models, we quantified biochemical mechanisms that control DA neurotransmission, including the kinetics of DA release, DAT uptake, and DA diffusion. Similar to a previous analysis of DA neurotransmission in awake mice (9), we found that three kinetic components effectively describe modulation of DA release over time: short-term facilitation, short-term depression, and long-term depression. We also analyzed the interactions of the synuclein family of proteins with DA release and mathematically quantified their effect. Our results support previous findings that α-Syn controls the short-term facilitation and long-term depression of DA release. Furthermore, we identified a new role for β-Syn and/or γ-Syn in the long-term regulation of DAT uptake and DA release.

The computational models we present have only been tested for in vivo FSCV recordings but could be adapted to ex vivo recordings in the striatal slice. The rapid recovery time of evoked DA release in vivo of < 10 ms (21) allows the electrical stimulation of midbrain neurons at 50 Hz, providing the means to study short-term synaptic plasticity. In contrast, the recovery time between electrical stimuli in slice is ∼1 min (31), which is due in part to the lack of tonic DA neuron firing and the lack of other neuronal activity in slice except for the cholinergic interneuron (32). As such, single electrical pulses cannot be recorded in the striatal brain slice without an extended recovery time and rapid electrical pulse trains produce a negligible increase in DA release.

**Fig. 9.**
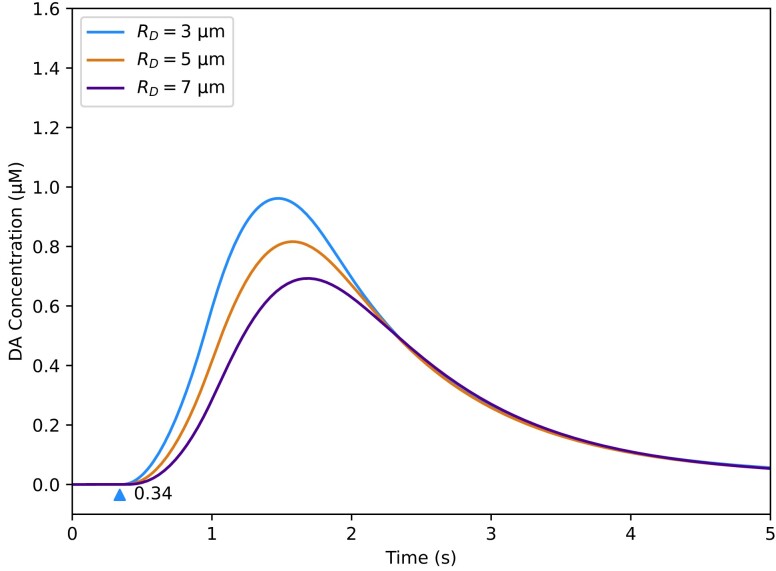
Effect of dead space radius on spatiotemporal uniform release model output. *R*_*D*_ is the parameter which controls the radius of the dead space in the model; 3 μm is the value used for fits presented in the Results section of the paper. Blue triangle indicates the start time of the electrical burst. The remaining model parameters are set to values in Fig. 3C (see Table S2).

Given that the three computational models produce similar fits to the data, they can be used without fundamentally altering the model's estimates for the DA release kinetics or DAT uptake. For fitting data with multiple burst stimuli such as the Repeated Burst protocol, users may achieve slightly improved computational performance using the Simple Uniform Release Model. However, it may be beneficial to use the spatiotemporal models if the FSCV experiments are performed in conjunction with other electrochemical or optical procedures to elucidate physical characteristics in the striatum. For example, it is possible to use amperometry to estimate the size of the dead space around the electrode, and the current dead space radius of 3 μm specified for Figs. 3–8 was derived from an amperometric study (10). As the dead space may vary from experiment to experiment which will alter the DA kinetics measured by the carbon-fiber electrode (Fig. 9), using amperometry and FSCV together can enable the dead space dimensions to be computed from amperometry data and used in the spatiotemporal models to fit FSCV data.

Additionally, nonelectrochemical DA recording methods such as optical reporters or false fluorescent neurotransmitters (FFNs) can be used to directly visualize the distribution of active and silent release sites in the striatum, which would make the Spatiotemporal Discrete Release Model particularly beneficial compared to other models. Currently, the distance between two adjacent release sites in this model for the fits presented in Figs. 3–8 is set to 6 μm based on numbers derived from FFN imaging reported by Pereira et al. (33) but assumes that the release sites are spaced at equal intervals. In theory, if the FSCV experiments are performed in conjunction with FFN or optical reporter experiments, the position and distance of release sites can be adjusted to any location according to the experiment. While our analysis of FSCV provides genuine measures of DA concentration, in the future, it may be possible to adapt the models and describe kinetics of the change in fluorescence (Δ*F*/*F*) data from fluorescent reporters, as many labs have access to fiber photometry.

The cylindrical diffusion of DA implemented in the spatiotemporal models may be particularly useful for modeling DA release under the influence of DAT-inhibiting drugs including amphetamine, cocaine (34), and nomifensine (35). The diameter of the cylinder is set to 100 μm for the fits presented in Figs. 3–8, less than the actual mouse striatum diameter of 2–3 mm (36). Under typical circumstances, distant release sites do not contribute to the final FSCV trace because DAT will remove distally released DA before it diffuses to the electrode. However, if DAT is at low levels or inhibited through pharmacological methods, the computed diameter of the striatum can be adjusted to capture DA diffusion in the extracellular space from distant release sites and account for the increase in DA concentration at the electrode.

With multiple adjustable parameters in the computational models that can be used to alter the outputs, it is theoretically possible to generate multiple sets of “best-fit” solutions that provide close fits to the FSCV data. In practice, however, the range of solutions is limited by the biological constraints applied to each parameter. For example, the affinity/binding constant of DAT (*K*_*m*_) can theoretically be any nonnegative value, but a consensus in the research literature is for *K*_*m*_ = 0.2 μM under normal conditions in mice (8), so fits that deviate from this standard without a valid biological reason can be ignored. To assist with providing the best fits with our computational models, we implemented a statistical inference algorithm known as ADVI (19, 20) for curve fitting that can limit the ranges for each parameter to effectively factor in the biological constraints.

## Materials and methods

### Computational models

The three models presented in this paper were implemented in Python, and the detailed discretization schemes used in the implementation can be found in the Supplementary Information. We used a combination of ordinary differential equations (ODEs), partial differential equations (PDEs), and time-series functions to implement these models.

#### Stimulation pattern

The function *S* is used to compute the electrical bursts applied from the stimulating bipolar electrode in FSCV experiments with the Heaviside theta function *θ*:


(1) $$S(t)=\underset{i}{\sum }\;\theta (t-t_{i})\theta \left(t_{i}+\frac{NP}{f}-t\right).$$

In this step function, *S*(*t*) = 1 denotes a time where an electrical burst is present, and *S*(*t*) = 0 denotes a time where an electrical burst is absent. The variable *t*_*i*_ denotes the start time for each burst, *NP* is the number of pulses in each burst, and *f* is the stimulus frequency (in Hz). Due to the increased lag time introduced by spatial diffusion in the dead space, *t*_*i*_ is modeled earlier in the STUR and STDR models compared to the SUR model.

#### Plasticity kinetics

The kinetics of stimulation-dependent DA release *A* is computed as the product of individual kinetic components *H*_*j*_, revised from Montague et al. (9):


(2) $$A(t)=\overset{N=3}{\underset{\;j=1}{\prod }}\;H_{j}(t),$$

(3) $$\frac{dH_{j}}{dt}=fp_{j}H_{j}S+(1-S)\frac{1-H_{j}}{\tau_{j}}.$$

During stimulus periods when *S*(*t*) = 1, a multiplicative plasticity factor *p*_*j*_ is applied to each kinetic component; positive values for *p*_*j*_ produce facilitation and negative values for *p*_*j*_ produce depression. During interstimulus periods when *S*(*t*) = 0, the kinetic components decay toward the equilibrium value of 1 based on the time constant *τ*_*j*_ until the next electrical burst is triggered. An example of the individual kinetic components for short-term facilitation, short-term depression, and long-term depression is shown in Fig. S2.

#### DA released into the striatum

Using the plasticity kinetics *A* and stimulation pattern *S* defined above, the DA released in the striatum [*DA*]_*S*_ is computed as the net difference between the concentration of DA released by electrical stimulation into the striatum and removed through DAT uptake. Each model implements [*DA*]_*S*_ differently based on the different temporal and spatial characteristics.

In the Simple Uniform Release Model, [*DA*]_*S*_ is implemented as an ODE that computes the change in concentration over time:


(4) $$\frac{d{\left[DA\right]}_{S}}{dt}=DA_{P}IfSAL-\frac{V_{m}{\left[DA\right]}_{S}}{{\left[DA\right]}_{S}+K_{m}}.$$

*DA*_*P*_ is the amount of DA released per milliamp (mA), and *I* is the electrical stimulus current in mA, such that *DA*_*P*_*I* is the concentration of DA released per pulse. The linear relationship established between DA release and stimulation current with *DA*_*P*_*I* can be found in FSCV experiments that apply electrical stimulation between 0.3 and 0.6 mA (Fig. 2B). *V*_*m*_ and *K*_*m*_ are the maximal velocity and affinity constant of DAT uptake (37), modeled using first-order Michaelis–Menten kinetics (38).

In the Spatiotemporal Uniform Release Model, [*DA*]_*S*_ is implemented as a PDE that simulates spatiotemporal diffusion and assigns a physical dimension to the dead space in brain tissue:


(5) $$\frac{\partial [DA]}{\partial t}=D\left(\frac{\partial^{2}[DA]}{\partial R^{2}}+\frac{1}{R}\frac{\partial [DA]}{\partial R}\right)+\eta \left(DA_{P}IfSA-\frac{V_{m}[DA]}{[DA]+K_{m}}\right),$$

(6) $$\eta (R)=\theta ((R_{L}-R_{D})-R),$$

(7) $$[DA{]}_{S}(t)=[DA](R=R_{L},t).$$

The striatum is modeled as a cylinder with uniformly distributed release sites, and the carbon-fiber electrode and dead space are positioned in the center of the striatum. The diffusion of DA in the striatum is simulated as isotropic diffusion using radial coordinates as described by John Crank (39), where *R* signifies the distance along the radius of the cylinder and *D* = 240 μm^2^/s is the diffusion coefficient of DA in the brain accounting for tortuosity (40). The dead space *η* is computed using the Heaviside theta function *θ*, where *R*_*L*_ is the radius of the cylinder and *R*_*D*_ is the radius of the dead space. The area covered by the dead space does not include DA release and DAT uptake due to the damaged tissue (10).

In the Spatiotemporal Discrete Release Model, [*DA*]_*S*_ is implemented nearly identically to the Spatiotemporal Uniform Release Model, except for the inclusion of an additional variable *P* to simulate spatially discrete release sites instead of a continuous release area:


(8) $$\frac{\partial [DA]}{\partial t}=D\left(\frac{\partial^{2}[DA]}{\partial R^{2}}+\frac{1}{R}\frac{\partial [DA]}{\partial R}\right)+\eta \left(DA_{P}IfSAP-\frac{V_{m}[DA]}{[DA]+K_{m}}\right),$$

(9) $$P(R)=\underset{i}{\sum }\;\delta (R-R_{i}).$$

*P* uses the Dirac delta function *δ* to simulate multiple “point” sources of release in the cylinder, with *R*_*i*_ specifying the location of each point along the radius, and each point source is a concentric cylinder due to the radial diffusion. Depending on the distribution of release points, the estimates for *DA*_*P*_ can be higher compared to the Simple Uniform Release and Spatiotemporal Uniform Release models (see Table 1); sparser distributions lead to higher release values from each individual point source.

#### DA measured at the electrode

Finally, the measurement of DA at the electrode is implemented as a coupled ODE system to model the temporal response and electrochemical adsorption that occur with the carbon-fiber electrode in FSCV experiments:


(10) $$\frac{d{\left[DA\right]}_{E}}{dt}=k_{S}[DA{]}_{S}-k_{E}[DA{]}_{E}+k_{\Gamma }\Gamma_{DA},$$

(11) $$\frac{d\Gamma_{DA}}{dt}=k_{1}^{ads}[DA{]}_{E}-k_{2}^{ads}[DA{]}_{E}\Gamma_{DA}-k_{3}^{ads}\Gamma_{DA}.$$

[*DA*]_*E*_ computes the DA diffusing from the extracellular space based on the value computed for [*DA*]_*S*_ minus the DA leaving the electrode due to the reflective surface in FSCV experiments, plus the residual DA that occurs due to adsorption on the electrode. *k*_*S*_ is the rate transfer of DA moving from the striatum toward the electrode, *k*_*E*_ is the rate transfer of DA moving away from the electrode, and $k_{\Gamma }=1$ is the rate transfer of DA adsorbing to the electrode. The adsorption Γ_*DA*_ is modeled as the concentration of DA that adsorbs to the electrode minus the concentration of DA that desorbs from the electrode, and it is a modified version of an equation presented by Bath et al. (17). The adsorption kinetic $k_{1}^{ads}$ controls the amount of DA that adheres to the electrode, and the desorption kinetics $k_{2}^{ads}$ and $k_{3}^{ads}$ control the amount of residual DA on the electrode that falls off. The full derivation for Equation (11) is provided in the Supplementary Information.

### Curve fitting

We implemented a statistical inference algorithm for curve fitting known as ADVI (19, 20) to assist with optimizing the parameters in our computational models and computing the best fit to the data. ADVI differs from conventional curve fitting algorithms used for parameter estimation in a few key aspects. First, ADVI can define constraints for each parameter estimate within a certain range of values, which is especially important for the models as it ensures biologically reasonable values that can still closely fit the data. Second, ADVI returns mean and standard deviation estimates for each parameter upon optimization rather than a singular point value; the standard deviation provides a measure of “uncertainty” for each parameter estimate and shows how much it could potentially vary while still explaining the data well. Third, ADVI is more efficient as it is a gradient-based optimization approach that can compute the optimization step in one pass for each iteration, in contrast to traditional nonconvex optimization approaches which require repeated numerical integration or Monte Carlo sampling.

The algorithm works by transforming the parameters from the original latent variable space to the unconstrained latent variable space. It then uses both the unconstrained and original parameters to compute an objective called the evidence lower bound (ELBO), comprised of the expected log joint density, the log Jacobian of the transformation, and the entropy of the unconstrained parameters (which are drawn from a Gaussian distribution) (19). The gradients of the unconstrained parameters with respect to the ELBO are used to optimize the original parameters, and the algorithm iterates until the parameters converge. Because the gradients can be computed efficiently using automatic differentiation, variational inference is typically faster than other statistical inference methods like Markov Chain Monte Carlo (MCMC) (41).

Applying ADVI requires adapting the ODE model into a probabilistic model to compute the expected log joint density for the ELBO, comprised of the log-prior and log-likelihood. To calculate the log-prior, the free parameters in the ODE model (*V*_*m*_, [*DA*]_*P*_, *p*_*j*_, *τ*_*j*_, *k*_*S*_, *k*_*E*_, $k_{1}^{ads}$, $k_{2}^{ads}$, and $k_{3}^{ads}$) are drawn from independent Gaussian distributions. For the log-likelihood, the ODE's solution at a given state ${\hat{C}}_{t}$ is computed by integrating [*DA*]_*E*_ over time, shown in Equation (12). ${\hat{C}}_{t}$ is then assigned as the mean of a Gaussian distribution to compute the log-likelihood (with the standard deviation *v* set to 0.05), and the corresponding state in the real data *C*_*t*_ is drawn from that distribution, as in Equation (13):


(12) $${\hat{C}}_{t}=\int_{0}^{t}\;[DA{]}_{E}(t)dt,$$

(13) $$C_{t}∼N({\hat{C}}_{t},v).$$

### In vivo recordings

To validate the computational models, we used previously published in vivo FSCV traces of DA release from WT and transgenic mice (21). Briefly, male or female mice were anesthetized with isoflurane (induction 2.5%, maintenance 0.8–1.4% in O_2_, 0.35 L/min), placed on a heating pad, and head-fixed on a stereotaxic frame. A craniotomy was performed to insert a 22G bipolar stimulating electrode (P1 Technologies) in the ventral midbrain to trigger electrical pulse trains and a carbon-fiber microelectrode (5 μm diameter, ∼150 μm length) in the dorsal striatum to measure evoked DA release; detailed stereotaxic coordinates and procedures can be found in the publication. The depth of the stimulating electrode was adjusted between 4 and 4.5 mm for maximal DA release. To conduct FSCV, a triangular voltage waveform (−450 to +800 mV at 294 mV/ms versus Ag/AgCl reference electrode) was applied to the carbon-fiber electrode at a 10 Hz sampling rate (or every 100 ms). The signals measured at the electrode were converted from analog to digital signals using a 16-bit data acquisition interface and subsequently recorded using IGOR Pro software. The electrical pulse trains were delivered to the stimulating electrode at a constant current of 400 μA using a stimulus isolator and a pulse generator. The stimulation protocols used in the experiments are explained in the Results. The carbon-fiber microelectrodes were calibrated in artificial cerebrospinal fluid using known concentrations of DA.

## Supplementary Material

pgad044_Supplementary_Data

## Data Availability

The software implementation of the models is available in a public repository on GitHub at https://github.com/DSulzerLab/DARELA.

## Preprints

This manuscript was posted on a preprint server: https://doi.org/10.1101/2022.05.04.490695.
