# Supplementary material for: Computational models of dopamine release measured by fast scan cyclic voltammetry in vivo
Source: PNAS Nexus. 2023 Feb 10;2(3):pgad044. doi: 10.1093/pnasnexus/pgad044 (PMC10003750; doi:10.1093/pnasnexus/pgad044)
Supplement: pgad044_Supplementary_Data [file pgad044_supplementary_data.pdf]

Model Implementation: The differential equations in the models were integrated using finite-difference discretization schemes. A finite-difference scheme discretizes a function into a grid by partitioning the temporal and spatial dimensions into evenly-spaced time points and space points, and the derivatives are computed at each time and space point to approximate the solution. In the Simple Uniform Release Model which is only integrated over time, the time step interval $\Delta t = \frac{1}{f}$, with f as the stimulation frequency in the FSCV experiments (50 Hz in the current data). In the spatiotemporal models, the time step $\Delta t = \frac{(\Delta R)^2}{2D}$ is computed based on the space step and the diffusion coefficient[1], where the space step ΔR is set to 1 µm and the diffusion coefficient for DA D is set to 240 µm^2/s based on the literature[2].

The ordinary differential equations (ODEs) in the computational models were discretized using the forward Euler scheme:

$$H_j(t + \Delta t) = H_j(t) + \Delta t \left[f p_j H_j(t) S(t) + \{1 - S(t)\} \frac{1 - H_j(t)}{\tau_j} \right] \quad \text{(S1)}$$

$$[DA]_E(t + \Delta t) = [DA]_E(t) + \Delta t [k_S [DA]_S(t) - k_E [DA]_E(t) + k_\Gamma \Gamma_{DA}(t)] \quad \text{(S2)}$$

$$\Gamma_{DA}(t + \Delta t) = \Gamma_{DA}(t) + \Delta t \left[k_1^{ads} [DA]_E(t) - k_2^{ads} [DA]_E(t) \Gamma_{DA}(t) - k_3^{ads} \Gamma_{DA}(t) \right] \quad \text{(S3)}$$

$$[DA]_S(t + \Delta t) = [DA]_S(t) + \Delta t \left[DA_P I f S(t) A(t) L - \frac{V_m [DA]_S(t)}{[DA]_S(t) + K_m} \right] \quad \text{(S4)}$$

For the partial differential equation (PDE) in the Spatiotemporal Uniform Release Model (Equation (S5)) and the Spatiotemporal Discrete Release Model (Equation (S6)), the cylindrical diffusion discretization was adapted from Venton et al.[1] using an explicit finite-difference method, and the DA release and reuptake were adapted from the ODE discretization schemes:

$$[DA](t + \Delta t, R) = [DA](t, R) + C_D + \eta(R)[DA_P I f S(t) A(t) - M] \Delta t \quad \text{(S5)}$$

$$[DA](t + \Delta t, R) = [DA](t, R) + C_D + \eta(R)[DA_P I f S(t) A(t) P(R) - M] \Delta t \quad \text{(S6)}$$

$$C_D = D \left[\frac{R}{2R - 1} [DA](t, R + \Delta R) + \frac{R - 1}{2R - 1} [DA](t, R - \Delta R) - [DA](t, R) \right] \Delta t \quad \text{(S7)}$$

$$M = \frac{V_m [DA](t, R)}{[DA](t, R) + K_m} \quad \text{(S8)}$$

DA Adsorption: To model the electrochemical adsorption of DA, we created a differential equation that computes an effective DA concentration $\Gamma_{[DA]}$, dependent on the concentration of DA measured by the

electrode $[DA]_E$ (see Equation 10 of the Methods section). The number of molecules adsorbed by the electrode over time N_{ads} can be written as:

$$\frac{dN_{ads}}{dt} = [DA]_E \frac{l}{t_1}(\Sigma_0 - \sigma N_{ads}) - \frac{N_{ads}}{t_2} \quad (S9)$$

l is the width of the layer of molecules that are in contact with the electrode, Σ_0 is the total surface area of the electrode, and σ is the surface area occupied by one molecule, such that $\Sigma = \Sigma_0 - \sigma N_{ads}$ is the free surface of the electrode. t_1 and t_2 are time constants that control the adsorption and desorption time, respectively. The term $[DA]_E l\Sigma$ represents the number of molecules near the free surface of the electrode available for adsorption. At the microscopic level, this equation originates from the observation that the electrical current measured by the electrode results from the number of charges that reach the electrode (which is approximated by the number of DA molecules that are in proximity of the free surface of the electrode over time) minus the number of particles that get adsorbed N_{ads} by the electrode.

Recasting Equation $(S9)$ in terms of concentration can be achieved by dividing all terms by $V = Bl$, with B having the dimensions of a surface:

$$\frac{d\Gamma_{[DA]}}{dt} = k_1^{ads}[DA]_E - k_2^{ads}[DA]_E\Gamma_{[DA]} - k_3^{ads}\Gamma_{[DA]} \quad (S10)$$

where $\Gamma_{[DA]} = \frac{N_{ads}}{Bl}$, $k_1^{ads} = \frac{\Sigma_0}{Bt_1}$, $k_2^{ads} = \frac{\sigma l}{t_1}$, and $k_3^{ads} = \frac{1}{t_2}$. This equation is equivalent to Equation 11 in the Methods section and used in the computational models. As shown in this derivation, Equation $(S10)$ can be obtained from Equation $(S9)$ using a few straightforward calculations by grouping and renaming the parameters.

References

1. Venton, B. J. *et al.* Real-time decoding of dopamine concentration changes in the caudate–putamen during tonic and phasic firing. *Journal of Neurochemistry* **87**, 1284–1295 (2003).
2. Nicholson, C. & Rice, M. E. Diffusion of ions and transmitters in the brain cell microenvironment. in *Volume Transmission in the Brain: Novel Mechanisms for Neural Transmission* 279–294 (Raven Publishing, 1991).

Supplemental Figures and Tables

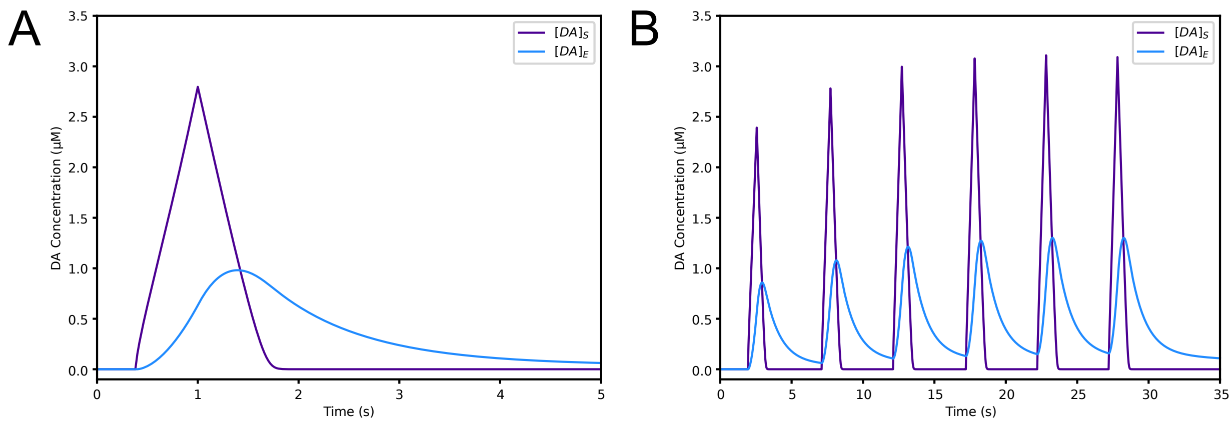

Figure S1: DA Released in Striatum ($[DA]_S$) vs. DA Measured at Electrode ($[DA]_E$). **A**, Single Burst protocol simulations with Simple Uniform Release Model for WT mice. **B**, Repeated Burst protocol simulations with Simple Uniform Release Model for WT mice. $[DA]_S$ (purple lines) models the release, reuptake, and diffusion of DA into the striatum accounting, while $[DA]_E$ (blue lines) accounts for DA release with the dead space, adsorption kinetics, and electrode temporal responses which affect the final trace measured in FSCV. Model parameters were set to values in Figure 3A and Figure 3B; see Table 1 and Table S1 for complete details.

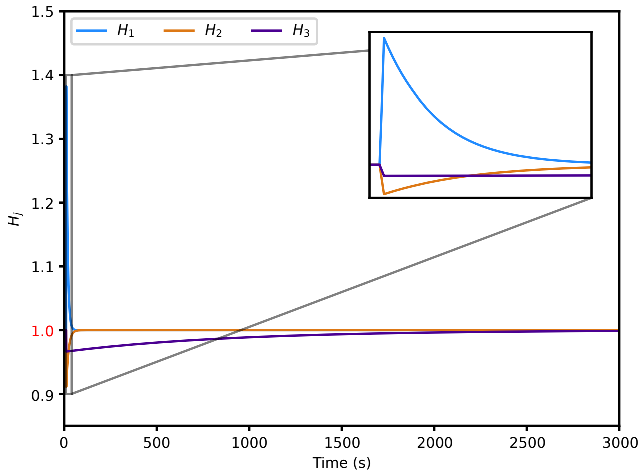

<u>**Figure S2:** Example of Individual Kinetic Components (H_j) in DA Computational Models.</u> H_1 (blue line) is short-term facilitation, H_2 (orange line) is short-term depression, and H_3 (purple line) is long-term depression. All three kinetics decay towards the equilibrium value of 1.0 (in red). Inset: individual kinetic components from 9 – 40 seconds, with 50 Hz, 30 pulse stimulation burst simulated at 10s. Plasticity factor and time constant values for each kinetic component were set to values from WT sweep 1 mice; see Table 1. The product of the three kinetic components determines the overall facilitative/depressive effect on the concentration of dopamine release. Refer to Equation 3 in the Methods section.

<u>Table S1:</u> <u>Best Fit Parameters of Simple Uniform Release Model.</u> Across all figures, I = 0.4 mA, f = 50 Hz, NP = 30 pulses, and L = 0.9.

	DA_P (μM/mA)	V_m (μM/s)	K_m (μM)	k_S (s^{-1})	k_E (s^{-1})	k_1^{ads} (s^{-1})	k_2^{ads} (s^{-1})	k_3^{ads} (s^{-1})
**Fig 3A**	0.420	4.8	0.2	0.9	1.05	0.035	0.140	0.090
**Fig 3B**	0.395	4.8	0.2	0.9	0.90	0.035	0.140	0.090
**Fig 4A**	0.305	3.2	0.2	0.9	1.00	0.035	0.140	0.090
**Fig 4B**	0.280	3.2	0.2	0.9	0.90	0.045	0.140	0.090
**Fig 5A**	0.460	4.8	0.2	0.9	1.00	0.040	0.140	0.090
**Fig 5B**	0.450	4.8	0.2	0.9	0.90	0.070	0.037	0.070
**Fig 6A**	0.320	3.2	0.2	0.9	1.00	0.035	0.140	0.090
**Fig 6B**	0.295	3.2	0.2	0.9	0.90	0.065	0.050	0.070
**Fig 7A**	0.460	4.8	0.2	0.9	1.00	0.055	0.020	0.075
**Fig 7B**	0.450	4.8	0.2	0.9	0.90	0.040	0.050	0.070
**Fig 8A**	0.506	5.6	0.2	0.9	1.00	0.035	0.020	0.075
**Fig 8B**	0.467	5.6	0.2	0.9	0.90	0.050	0.020	0.075

Table S2: Best Fit Parameters of Spatiotemporal Uniform Release Model. Across all figures, I = 0.4 mA, f = 50 Hz, NP = 30 pulses, R_L = 50 µm, and R_D = 3 µm.

	DA_P (µM/mA)	V_m (µM/s)	K_m (µM)	k_S (s^{-1})	k_E (s^{-1})	k_1^{ads} (s^{-1})	k_2^{ads} (s^{-1})	k_3^{ads} (s^{-1})
**Fig 3C**	0.422	4.8	0.2	0.9	1.05	0.025	0.140	0.090
**Fig 3D**	0.400	4.8	0.2	0.9	0.90	0.035	0.140	0.090
**Fig 4C**	0.310	3.2	0.2	0.9	1.00	0.035	0.140	0.090
**Fig 4D**	0.290	3.2	0.2	0.9	0.90	0.045	0.140	0.090
**Fig 5C**	0.460	4.8	0.2	0.9	1.00	0.020	0.140	0.090
**Fig 5D**	0.443	4.8	0.2	0.9	0.90	0.070	0.037	0.070
**Fig 6C**	0.315	3.2	0.2	0.9	1.00	0.035	0.140	0.090
**Fig 6D**	0.295	3.2	0.2	0.9	0.90	0.065	0.050	0.070
**Fig 7C**	0.460	4.8	0.2	0.9	1.00	0.035	0.020	0.075
**Fig 7D**	0.450	4.8	0.2	0.9	0.90	0.040	0.050	0.070
**Fig 8C**	0.505	5.6	0.2	0.9	1.00	0.020	0.020	0.075
**Fig 8D**	0.467	5.6	0.2	0.9	0.90	0.050	0.020	0.075

<u>Table S3:</u> <u>Best Fit Parameters of Spatiotemporal Discrete Release Model.</u> Across all figures, I = 0.4 mA, f = 50 Hz, NP = 30 pulses, R_L = 50 µm, and R_D = 3 µm.

	DA_P (µM×µm/mA)	V_m (µM/s)	K_m (µM)	k_S (s^{-1})	k_E (s^{-1})	k_1^{ads} (s^{-1})	k_2^{ads} (s^{-1})	k_3^{ads} (s^{-1})
**Fig 3E**	2.43	4.8	0.2	0.9	1.05	0.025	0.140	0.090
**Fig 3F**	2.30	4.8	0.2	0.9	0.90	0.035	0.140	0.090
**Fig 4E**	1.79	3.2	0.2	0.9	1.00	0.035	0.140	0.090
**Fig 4F**	1.70	3.2	0.2	0.9	0.90	0.045	0.140	0.090
**Fig 5E**	2.65	4.8	0.2	0.9	1.00	0.020	0.140	0.090
**Fig 5F**	2.55	4.8	0.2	0.9	0.90	0.070	0.037	0.070
**Fig 6E**	1.80	3.2	0.2	0.9	1.00	0.035	0.140	0.090
**Fig 6F**	1.70	3.2	0.2	0.9	0.90	0.065	0.050	0.070
**Fig 7E**	2.63	4.8	0.2	0.9	1.00	0.055	0.020	0.075
**Fig 7F**	2.55	4.8	0.2	0.9	0.90	0.040	0.050	0.070
**Fig 8E**	2.77	5.6	0.2	1.0	1.00	0.020	0.020	0.075
**Fig 8F**	2.65	5.6	0.2	0.9	0.90	0.050	0.020	0.075
